# Examining the association between aggression and suicide attempts among army soldiers

**DOI:** 10.1017/S0033291724002460

**Published:** 2024-11

**Authors:** Alison Krauss, Ashley L. Greene, Emily R. Edwards, Marianne Goodman

**Affiliations:** 1 VA Veterans Integrated Service Network (VSIN) 17 Center of Excellence for Research on Returning War Veterans, Waco, TX, USA; 2 Central Texas Veterans Health Care System, Temple, TX, USA; 3 VA VISN 2 Mental Illness Research, Education, and Clinical Center, Bronx, NY, USA; 4 Department of Psychiatry, Yale School of Medicine, New Haven, CT, USA; 5 Department of Psychiatry, Icahn School of Medicine at Mt. Sinai, New York, NY, USA

**Keywords:** alcohol use, firearms, military, physical aggression, social networks, suicide risk, verbal aggression

## Abstract

**Background:**

Suicide is a major concern among active-duty military personnel. Aggression represents a salient risk factor for suicide among civilians, yet is relatively understudied among military populations. Although several theories posit a relation between aggression and suicide with putative underlying mechanisms of social isolation, access to firearms, and alcohol use, researchers have yet to test these potential mediators. This study uses rich, longitudinal data from the Army Study to Assess Risk and Resilience (STARRS) Pre/Post Deployment Study (PPDS) to examine whether aggression longitudinally predicts suicide attempts and to identify mediators of this association.

**Methods:**

Army soldiers (*N* = 8483) completed assessments 1 month prior to deployment and 1, 2–3, and 9–12 months post-deployment. Participants reported on their physical and verbal aggression, suicide attempts, social network size, firearm ownership, and frequency of alcohol use.

**Results:**

As expected, pre-deployment aggression was significantly associated with suicide attempts at 12-months post-deployment even after controlling for lifetime suicide attempts. Social network size and alcohol use frequency mediated this association, but firearm ownership did not.

**Conclusions:**

Findings further implicate aggression as an important suicide risk factor among military personnel and suggest that social isolation and alcohol use may partially account for this association.

Suicide rates in the military have steadily increased in the past decade, with most recent estimates suggesting a rate of approximately 25 per 100 000 active-duty military personnel per year (Department of Defense, 2022). Though sparse, existing research suggests aggression may be a strong predictor of suicide attempts among military personnel (Schafer et al., 2022). Unfortunately, aggression is also common among military personnel: approximately 30% of veterans report engaging in physical aggression (e.g. behaviors intended to cause physical harm) in a given month and 10% report engaging in severe violence (e.g. use of a weapon against another) in a given year (MacManus et al., 2015). To date, driving factors underlying the association between service member aggression and suicide remain largely unknown. Using rich, longitudinal data from the Army Study to Assess Risk and Resilience in Service Members (STARRS) Pre/Post Deployment Study (PPDS), the current study examines whether aggression predicts suicide attempts among Army soldiers and explores potential mediators of this association.

## Aggression as a suicide risk factor

Substantial research highlights aggression – defined as behaviors intended to harm another person who is motivated to avoid harm (Allen & Anderson, 2017) – as a salient risk factor for suicide among civilians (Franklin et al., 2017; Schafer et al., 2022). Longitudinal studies, for example, find that aggression tends to temporally precede suicide attempts and is associated with greater medical severity of attempts (Gvion, 2018; Oquendo et al., 2021). Comparable research among service members and veterans is sparse. Cross-sectional studies suggest aggression is correlated with suicidal ideation (McGlade et al., 2021) and seems to co-occur with suicide attempts (Elbogen et al., 2018). Among veterans in a posttraumatic stress disorder (PTSD) treatment program, for example, endorsement of both aggression and past suicide attempts was more common than endorsement of only past suicide attempts (Watkins, Sippel, Pietrzak, Hoff, & Harpaz-Rotem, 2017). Similarly, in active-duty samples, service members reporting any aggression were more likely to endorse suicidal ideation than service members reporting no aggression (Start, Allard, Adler, & Toblin, 2019).

In a recent meta-analytic review of suicide risk factors in military personnel, Schafer et al. (2022) identified just five longitudinal studies of aggression and suicide among service members and no longitudinal studies of this association among veterans. Nonetheless, pooled estimates from these five studies identified aggression as the strongest predictor of suicide death among service members (Schafer et al., 2022). Relatedly, results from the STARRS Longitudinal Study suggest a history of physical assault against others and a childhood history of bullying or threatening each independently predict post-discharge suicide attempts in service members even after controlling for dozens of other potential predictors (Kearns et al., 2023). The relative dearth of research on the link between aggression and suicide stands in stark contrast to evidence suggesting generally elevated rates of aggression among military personnel.

## Mechanisms of the aggression – suicide association

In addition to the scarcity of research linking aggression to suicide among military personnel, few, if any, studies have identified mechanisms of this association. Many theories of suicide provide potential explanations for how aggression increases suicide risk. For instance, the Interpersonal Theory of Suicide (IPT; Van Orden et al., 2010) and Three-Step Theory of Suicide (3ST; Klonsky & May, 2015) suggest that greater numbers of interpersonal relationships may buffer against suicide risk, whereas social isolation contributes to the development of suicide attempts (Calati et al., 2019; Van Orden et al., 2010). Aggressive behaviors tend to strain important relationships; correspondingly, aggression is consistently associated with greater social isolation and interpersonal conflict (Hammett, Lavner, Karney, & Bradbury, 2021; Rynar & Coccaro, 2018). Aggression researchers speculate that these relational consequences of aggression may help explain the association between aggression and suicide (Conner, Duberstein, Conwell, & Caine, 2003; Hartley, Pettit, & Castellanos, 2018). Nevertheless, the potential for social isolation to serve this mediating role has not been empirically tested.

IPT and 3ST also note the importance of capability to attempt suicide – factors that decrease barriers to engaging in suicide-related behaviors (Klonsky & May, 2015; Van Orden et al., 2010). Within this framework, aggression may be conceptualized as contributing to capability for suicide. Indeed, aggression is positively associated with firearm ownership (a form of practical capability) in both veteran (Heinz, Cohen, Holleran, Alvarez, & Bonn-Miller, 2016) and civilian samples (Clare et al., 2021; Sanchez, Jaguan, Shaikh, McKenney, & Elkbuli, 2020). Firearms represent a particularly lethal method of suicide: the estimated fatality rate of attempts made with firearms is around 90% compared to around 50% for attempts by hanging, 28% for attempts by jumping, and 2% for attempts by drug overdose (Conner, Azrael, & Miller, 2019). In addition, military personnel are more likely to use firearms in a suicide attempt compared to civilians (Gromatsky et al., 2022); almost 70% of service members who make a suicide attempt do so with a firearm (Department of Defense, 2022). It may be that aggression is associated with higher rates of firearm ownership, which in turn increases risk for suicide attempts.

Lastly, problematic alcohol use may mediate the association between aggression and suicide attempts. Alcohol use represents a consistent risk factor for suicide attempts among both civilians (Poorolajal, Haghtalab, Farhadi, & Darvishi, 2016) and military personnel (Bohnert, Ilgen, Louzon, McCarthy, & Katz, 2017). Alcohol use is also associated with aggression, with some evidence suggesting problematic aggression often precedes alcohol use (Coccaro et al., 2016). Theoretical models of suicide suggest that alcohol use may mediate the association between aggression and suicide such that problematic aggression gives rise to chronic or severe alcohol use which, in turn, increases risk for suicide attempts (Conner et al., 2003; Conner & Ilgen, 2011). Again, however, this claim has not been empirically tested.

## Current study

The current study extends our understanding of aggression as a longitudinal risk factor for suicide attempts among military personnel. First, we aimed to replicate the previously observed longitudinal association between aggression and suicide attempts, thereby adding to the small but growing body of research suggesting aggression is associated with later suicide attempts. Second, we tested whether social isolation (defined by the size of one's social network), firearm ownership, and/or problematic alcohol use mediate the relation between aggression and suicide attempts. We examined these associations using the Army STARRS PPDS, a large dataset of deployed Army soldiers spanning four time points: pre-deployment (T0), 1-month post-deployment (T1), 2–3 months post-deployment (T2), and 9–12 months post-deployment (T3). The Army STARRS PPDS data are ideally suited to address the current research question as Army STARRS studies were specifically designed to examine risk and resilience factors in suicide behaviors of military personnel over time (Kessler et al., 2013a). We hypothesized that (1) aggression at T0 would predict suicide attempts at T3, while controlling for lifetime suicide attempts at T0, and that (2) social network size, firearm ownership, and problematic alcohol use at T2 would partially mediate the association between T0 aggression and T3 suicide attempts. We tested these hypotheses while controlling for history of major depressive disorder (MDD), history of PTSD, history of traumatic brain injury (TBI), highest level of education, and trauma exposure on deployment.

## Methods

### Participants

Soldiers from three Brigade Combat Teams were recruited for the PPDS approximately 1 month prior to a combat deployment to Afghanistan, with data collection occurring from 2012 to 2014. To be included in the current study, participants must have provided complete data on aggression and lifetime suicide attempts at the T0 survey, resulting in a sample of 8483 soldiers. The majority of soldiers identified as men (93.6%) with an average age of 26.06 years (s.d. = 6.09) at T0. Most soldiers identified as White (66.1%), followed by Hispanic/Latino (15.8%), Black/African American (12.8%), Asian (4.4%), Native American/Alaskan Native (3.1%), Native Hawaiian/Pacific Islander (2.2%), and ‘Other’ (9.4%); participants could have indicated more than one racial or ethnic identity. Approximately 7% of soldiers reported earning a GED or equivalent, 41.2% a high school diploma, 25.6% some post-high school education, 3.1% a technical school certificate or degree, 7.6% an associate degree, 10.2% a bachelor's degree, and 2.4% some graduate or professional education.

### Procedures

Soldiers were recruited, provided informed consent, and completed the T0 survey in a group setting approximately 1 month prior to deployment. T1 surveys were completed within 1 month of returning from deployment, T2 surveys completed 2–3 months post-deployment, and T3 surveys completed 9–12 months post-deployment. Further details about survey administration can be found elsewhere (Kessler et al., 2013a). Secondary data analysis for the current study was approved by the Institutional Review Board of the first author's institution.

### Measures

#### Aggression

Soldiers reported their aggression at T0 using four items derived from the Joint Mental Health Advisory Team 7 (Office of the Surgeon General, United States Army Medical Command et al., 2011). Participants indicated how frequently they had engaged in each item on a five-point scale ranging from 0 (*never*) to 4 (*very often*); no reference period was indicated. Items included: (1) yell, insult, swear, or threaten someone; (2) have a heated argument with someone; (3) get into a loud argument in public; (4) have a physical confrontation during an argument. Per prior research with this measure (Krauss et al., 2023), responses were summed with higher scores indicating greater engagement in aggressive behaviors; scores ranged from 0 to 16. Internal consistency in the current sample was adequate (*α* = 0.80).

#### Social networks

At T0 and T2, soldiers indicated the extent of their social network across four items developed by the Army STARRS research team. Participants were asked ‘How many people do you have in your personal life of the following sorts?’ with items including: (1) people you do things with, like watch TV together, go out for a drink or movie together, or play cards; (2) people who you feel really close to; (3) people who really care for you and would be there if you needed them; (4) family or friends who need you and rely on you for help when they need it. Participants responded on a 10-point scale ranging from 0 (*0 people*) to 9 (*31 or more people*). Items were averaged with higher scores representing a larger social network; scores ranged from 0 to 9. Internal consistency was *α* = 0.85 at T0 and *α* = 0.89 at T2.

#### Alcohol use

Participants reported on their frequency of alcohol use at T0 and T2 using the self-administered Composite International Diagnostic Interview Screening Scales (CIDI-SC; Kessler et al., 2013b). Soldiers were asked how often during the past 30 days they had five or more drinks of alcohol on the same day. Since frequency of alcohol use was measured with a single item, internal consistency could not be calculated.

#### Firearm ownership

Soldiers reported their firearm ownership at T0 and T2 using a single question: ‘How many guns in working condition do you have in your home (house, apartment, barracks) including handguns, rifles, and shotguns?’ Response options ranged from 0 (*0 guns*) to 6 (*11 or more guns*). This item is designed to assess respondents’ ownership of personal firearms rather than access to firearms available through their military service.

#### Suicide attempts

At T0, T2, and T3, soldiers reported on suicide attempts using a single item adapted from the Columbia Suicide Severity Rating Scale (Posner et al., 2011). At T0, soldiers reported on whether they had ever made a suicide attempt in their lifetime. T0 suicide attempts were coded with 1 representing one or more suicide attempts in their lifetime and 0 representing no lifetime history of suicide attempts. At T2, soldiers indicated whether they had made a suicide attempt since returning from deployment. At T3, soldiers who had completed T2 reported on suicide attempts since their T2 survey, and soldiers who did not complete T2 reported on suicide attempts since returning from deployment. This ensured that soldiers who completed T3, but not T2, had a report of suicide attempts across the same time period (since returning from deployment) as soldiers who completed both T2 and T3. Consistent with previous research using this measure (Chu et al., 2020), we combined T2 and T3 reports; the composite score was coded such that 1 represents endorsement of a suicide attempt since returning from deployment, and 0 represents no endorsement (we refer to this score as suicide attempts at T3).

#### Control variables

At T0, soldiers reported on their history of MDD using the CIDI-SC (Kessler et al., 2013b) and reported on their history of PTSD using a screening version of the PTSD Checklist (Wilkins, Lang, & Norman, 2011). Diagnoses were coded such that 1 represents a history of the diagnosis and 0 represents no history. Soldiers also completed seven items assessing whether they had ever experienced a head injury involving loss of consciousness or a lapse in memory. History of TBI was scored such that 1 represents the presence of TBI of at least mild severity (defined as a head injury resulting in loss of consciousness for less than 30 minutes or lapse in memory of less than 24 hours, per the American Congress for Rehabilitation Medicine; Silverberg et al., 2023), and 0 represents no history of TBI.

Since deployment represents a methodological control in the current study, we included trauma exposure while on deployment as a control variable. At T1, soldiers reported on their exposure to potentially traumatic events during deployment using the Army STARRS Combat Experiences Scale (CES; Sherman, Frye-Cox, & Lucier-Greer, 2023), adapted from the Deployment Risk and Resilience Inventory (Vogt et al., 2013). The CES includes 12 items rated on a 5-point scale from 0 (*never*) to 4 (*10* *+* *times*). Items assessed a variety of potentially traumatic experiences often seen in combat deployments, such as getting wounded, having a ‘close call,’ and having responsibility for the death of another. Items were averaged such that higher scores represented greater exposure on deployment; observed scores ranged from 0 to 3.9. Internal consistency in the current sample was *α* = 0.74. Previous research suggests good convergent validity of the CES, with CES total scores correlating positively with depressive and anxiety symptoms and negatively with coping ability (Sherman et al., 2023).

### Missing data analyses and data analytic plan

Missing data ranged from 0.72% (firearm ownership at T0) to 26% (social networks at T2). Table 1 presents demographic characteristics of participants with and without complete data. We imputed missing data values across all variables at all time points using missForest (Stekhoven & Bühlmann, 2012), an imputation method appropriate for non-parametric, mixed type (categorical or continuous) data. MissForest outperforms other imputation methods, such as multiple imputation by chained equations, when data are non-parametric and mixed-type (Stekhoven & Bühlmann, 2012). In addition to all main study variables, we also included age, gender, race, and ethnicity in the imputation model as these characteristics were related to missingness of study variables. We imputed reports of suicide attempts at T2 and T3 separately and then calculated the composite suicide attempts score after imputation. The random forest algorithm also allows for estimation of out-of-bag (OOB) error rates, providing an estimate of the convergence of imputation (OOB values close to 0 suggest excellent convergence while OOB values close to 1 suggest poor convergence). The OOB estimate was 0.25 for continuous variables and 0.17 for categorical variables, reflecting good convergence.

**Table 1.** Demographic characteristics by participants with complete and incomplete data

Characteristic		Complete data^a^ (*n* = 5349)	Incomplete data^b^ (*n* = 3134)
Mean age (s.d.)		26.05 (5.97)	26.09 (6.32)
Gender			
	Man	5045 (94.3%)	2820 (90.0%)
	Woman	304 (5.7%)	236 (7.5%)
Ethnicity			
	Hispanic/Latino/a	839 (15.7%)	516 (16.5%)
	Non-Hispanic/Latino/a	4520 (84.5%)	2416 (77.1%)
Race			
	White	3572 (66.8%)	1955 (62.4%)
	Black/African American	644 (12.0%)	444 (14.2%)
	Asian American	255 (4.8%)	118 (3.8%)
	Native American/Alaskan Native	156 (2.9%)	103 (3.3%)
	Native Hawaiian/Pacific Islander	125 (2.3%)	60 (1.91%)
	Other	512 (9.6%)	287 (9.2%)
Education			
	GED or equivalent	346 (6.5%)	238 (7.6%)
	High school diploma	2155 (40.3%)	1325 (42.3%)
	Some post-high school education	1432 (26.8%)	725 (23.1%)
	Technical school certificate/degree	326 (6.1%)	191 (6.1%)
	Associate degree	410 (7.7%)	234 (7.5%)
	Bachelor's degree	580 (10.8%)	279 (8.9%)
	Some graduate/professional education	100 (1.9%)	101 (3.2%)

a Complete data were defined as full data on aggression at T0, lifetime suicide attempts at T0, all mediators at T2, age, gender, ethnicity, race, and level of education.

b Rows within the Incomplete data column may not add to 3134 as some participants had missing data on demographic characteristics.

**Figure 1.**
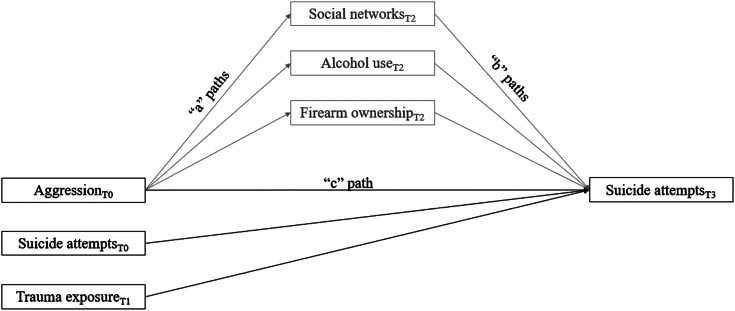
Proposed mediation model depicting the association between aggression and suicide attempts via social networks, alcohol use, and firearm ownership. Paths marked in black are tested in hypothesis 1 and paths marked in gray are tested in hypothesis 2.

After imputing missing data, we used path analyses through the R package lavaan (Rosseel, 2012) to examine our mediation hypotheses; Fig. 1 presents the hypothesized mediation model. To test hypothesis 1 (aggression at T0 would predict suicide attempts at T3), we entered aggression at T0, lifetime suicide attempts at T0, and the control variables as predictors and suicide attempts at T3 as the outcome. To test hypothesis 2 (social network size, firearm ownership, and frequency of alcohol use would mediate the association between aggression and suicide attempts), we entered each ‘a’ path (aggression at T0 predicting each mediator at T2) while controlling for each mediator at T0; this allowed us to examine the association between aggression at T0 and changes in mediators from T0 to T2. We then entered each ‘b’ path (mediators at T2 predicting suicide attempts at T3) and the ‘c’ path (aggression at T0 predicting suicide attempts at T3). We again controlled for the effect of lifetime suicide attempts at T0 and our control variables on suicide attempts at T3. We calculated indirect effects using the product of coefficient approach where we computed the product of each ‘a’ and ‘b’ path (Hayes, 2022). Across all models, we used diagonally weighted least squares estimators with robust standard errors; this estimator is appropriate for use with categorical or skewed outcome variables as it makes no assumptions about the underlying distribution of variables (Li, 2016).

## Results

### Descriptive statistics

Table 2 presents descriptive statistics and correlations between main study variables. Overall prevalence of lifetime suicide attempts at T0 (2.0%, *n* = 165) and suicide attempts at T3 (0.9%, *n* = 76) was low. On average, soldiers reported moderate levels of aggression and having about four individuals in their social network. Most soldiers reported owning 0 firearms at T0 and T2; approximately 30% reported owning at least one firearm at T0 and 36% at T2.

**Table 2.** Descriptive statistics and correlations between main study variables

	*1*	*2*	*3*	*4*	*5*	*6*	*7*	*8*	*9*	*10*	*11*	*12*	*M* (s.d.) *%* (*n*)
1. Aggression T0	–												2.96 (2.92)
2. Suicide T0	0.12*	–											2.0% (165)
3. Suicide T3	0.05*	0.10*	–										0.9% (76)
4. Social networks T0	−0.11*	−0.05*	−0.04*	–									4.11 (1.89)
5. Social networks T2	−0.09*	−0.05*	−0.04*	0.51*	–								3.92 (1.78)
6. Alcohol use T0	0.25*	0.03	0.01	0.01	0.02	–							1.88 (0.96)
7. Alcohol use T2	0.18*	0.03*	0.03*	0.02	0.00	0.56*	–						1.90 (1.01)
8. Firearm ownership T0	0.12*	−0.00	−0.01	0.01	−0.01	0.08*	0.07*	–					0.83 (1.53)
9. Firearm ownership T2	0.11*	−0.01	−0.02	0.01	0.01	0.09*	0.09*	0.77*	–				0.93 (1.59)
10. Trauma exposure T1	0.13*	−0.01	0.01	0.10*	0.08*	0.17*	0.17*	0.14*	0.14*	–			0.95 (0.59)
11. MDE at T0	0.32*	0.17*	0.07*	−0.16*	−0.14*	0.11*	0.09*	0.01	0.00	0.03*	–		9.4% (880)
12. PTSD at T0	0.32*	0.14*	0.06*	−0.12*	−0.10*	0.10*	0.07*	0.05*	0.03*	0.04*	0.39*	–	12.4% (1053)
13. TBI at T0	0.21*	0.04*	0.02	−0.02	−0.01	0.16*	0.12*	0.16*	0.16*	0.17*	0.14*	0.17*	62.8% (5324)

*Note.* Correlations between continuous variables are Pearson's *r*, correlations between binary variables are phi coefficients, and correlations between a continuous and a binary variable are point-biserial correlations. Percentages and sample sizes are the proportion of participants who endorsed that variable. Aggression scores ranged from 0 to 16; social network scores ranged from 0 to 9; alcohol use scores ranged from 1 to 5; firearm ownership scores ranged from 0 to 6.

* *p* < 0.01.

### Mediation analysis

To test our first hypothesis, we examined the association between aggression at T0 and suicide attempts at T3 while controlling for lifetime suicide attempts at T0 and our control variables. History of PTSD (*β* = 0.04, s.e. = 0.04, 95% CI −0.03 to 0.12, *p* = 0.25), history of TBI (*β* = 0.02, s.e. = 0.05, 95% CI −0.07 to 0.12, *p* = 0.62), education (*β* = −0.11, s.e. = 0.06, 95% CI −0.23 to 0.01, *p* = 0.07), and trauma exposure at T1–T3 (*β* = 0.01, s.e. = 0.05, 95% CI −0.09 to 0.10, *p* = 0.93) were not associated with suicide attempts at T3; we therefore removed these variables from the model. Aggression (*β* = 0.10, s.e. = 0.04, 95% CI 0.02–0.18, *p* = 0.01), lifetime suicide attempts (*β* = 0.10, s.e. = 0.02, 95% CI 0.05–0.14, *p* < 0.001), and history of MDD (*β* = 0.11, s.e. = 0.03, 95% CI 0.05–0.17, *p* = 0.001) were associated with suicide attempts at T3 such that greater levels of aggression, reporting a lifetime suicide attempt, and a history of MDD were positively associated with likelihood of reporting a suicide attempt at T3. This model accounted for 4.4% of the variance in suicide attempts at T3.

To test our second hypothesis, we added paths from aggression at T0 to each mediator at T2 while controlling for each mediator at T0 and paths from each mediator at T2 to suicide attempts at T3. Firearm ownership at T2 was not associated with suicide attempts at T3 (*β* = −0.08, s.e. = 0.05, 95% CI −0.18 to 0.02, *p* = 0.11), and firearm ownership did not mediate the association between aggression at T0 and suicide attempts at T3 (*β* = −0.01, s.e. = 0.01, 95% CI −0.01 to 0.01, *p* = 0.14). Thus, we removed firearm ownership from our model. The final model included aggression at T0, lifetime suicide attempts, history of MDD, and social networks and alcohol use frequency as potential mediators.

Table 3 presents results of the final mediation model. All direct effects (‘a’ paths and ‘b’ paths) were significant. Aggression at T0 was associated with both a smaller social network and greater alcohol use frequency at T2 after controlling for social network and alcohol use at T0. Aggression at T0, suicide attempts at T0, history of MDD, social networks at T2, and alcohol use frequency at T2 all evidenced direct effects on suicide attempts at T3 in predicted directions. Specifically, greater levels of aggression, reporting a lifetime suicide attempt at T0, a history of MDD, a smaller social network, and greater frequency of alcohol use were associated with reporting a suicide attempt at T3. Further, aggression at T0 demonstrated a significant indirect effect on suicide attempts at T3 via social networks at T2 and alcohol use frequency at T2. The full mediation model accounted for 6.2% of the variance in suicide attempts at T3, suggesting addition of social network and alcohol use frequency accounted for an additional 2% of the variance in T3 suicide attempts beyond T0 aggression.

**Table 3.** Direct and indirect effects of aggression on suicide attempts via social networks and alcohol use frequency

			95% CI
Path	*β* (s.e.)	LL	UL
Direct effects			
Aggression T0 → social networks T2	−0.02 (0.01)**	−0.04	−0.01
Social networks T0 → social networks T2	0.50 (0.01)**	0.50	0.52
Aggression T0 → alcohol use T2	0.04 (0.01)**	0.02	0.06
Alcohol use T0 → alcohol use T2	0.57 (0.01)**	0.55	0.58
Aggression T0 → suicide attempts T3	0.09 (0.04)*	0.01	0.17
Suicide attempts T0 → suicide attempts T3	0.10 (0.02)**	0.05	0.14
History of MDD T0 → suicide attempts T3	0.10 (0.03)**	0.03	0.16
Social networks T2 → suicide attempts T3	−0.09 (0.04)*	−0.16	−0.01
Alcohol use T2 → suicide attempts T3	0.09 (0.04)**	0.01	0.16
Indirect effects			
Aggression T0 → social networks T2 → suicide attempts T3	0.002 (0.001)	0.000	0.005
Aggression T0 → alcohol use T2 → suicide attempts T3	0.004 (0.002)*	0.001	0.001
Total effect	0.10 (0.04)*	0.02	0.18
*R*^2^	0.06		

*p < 0.05; **p < 0.01.

## Discussion

This study investigated the link between aggression and suicide attempts in active duty Army personnel and explored potential mediators of this association, including social networks, alcohol use frequency, and firearm ownership. Consistent with our first hypothesis, aggression predicted later suicide attempts while controlling for lifetime suicide attempts. Indeed, aggression continued to demonstrate a direct effect on suicide attempts in our full model while accounting for the effect of social networks and alcohol use frequency. These findings add to the sparse body of research implicating aggression as a longitudinal predictor of suicide attempts in service members (Schafer et al., 2022) and reinforce the notion that aggression is a valuable predictor of suicide attempts.

In addition to examining the direct association between aggression and suicide attempts among Army service members, the present study represents one of the first attempts to identify mechanisms of this association. In support of hypothesis 2, social networks and alcohol use frequency partially mediated the association between aggression and suicide attempts. Our findings support theories of aggression and suicide that speculate that lower social connection and frequent alcohol use may partially explain how aggression increases risk for suicide (Conner et al., 2003; Hartley et al., 2018). Specifically, it may be that problematic aggression leads to interpersonal conflict and subsequent loss of important social connections. Individuals who struggle with aggression may then experience significant social isolation and loneliness, which are strongly associated with suicidal ideation and attempts (Calati et al., 2019). Similarly, problematic aggression may give rise to frequent alcohol use, perhaps as a way to cope with adverse consequences of aggression or to manage aggressive impulses, which in turn may increase risk for suicide. These results underscore the potential utility of enhancing social support and addressing alcohol use to mitigate suicide risk in military settings.

Unexpectedly, firearm ownership did not mediate the association between aggression and suicide attempts. On the surface, this finding seems to diverge from prior work suggesting that access to firearms escalates suicide risk (Houtsma, Butterworth, & Anestis, 2018). On the other hand, there are many potential explanations for this unexpected finding. For instance, our measure of firearm ownership specifically asked about firearms in the home (house, apartment, barracks), as opposed to access to firearms both in and outside of the home. It may be that access to firearms outside of the home is important in understanding the association between aggression and suicide. This suggestion is in line with research indicating that individuals with access to firearms through their occupation are at increased risk for suicide compared to those without access (Milner, Witt, Maheen, & LaMontagne, 2017). Alternatively, it may be that firearm ownership is an important predictor of lethal suicide attempts but not of non-lethal suicide attempts. Given that all self-reported suicide attempts in the current study were non-lethal, our results may have underestimated the true association between firearm ownership and suicide attempts. Further research on the role of firearms in the link between aggression and suicide among military personnel is clearly warranted.

### Implications for future research and suicide prevention

The present study holds important implications for future research and suicide prevention efforts. For one, researchers should attempt to replicate our results among samples of service members and veterans who are undergoing the process of separating from service as this period is associated with loss of military social connections and a rise in suicide-related behavior (Sokol et al., 2021; U.S. Department of Veterans Affairs, Office of Mental Health and Suicide Prevention, 2023). Second, the fact that social networks and alcohol use *partially* mediated the association between aggression and suicide attempts suggests the presence of other mediating factors. Future research should examine whether other potential consequences of aggression (e.g. incarceration, loss of employment, mental health service utilization) impart increased risk for suicide among military personnel. Finally, further work should explore how shared mechanisms of aggression and suicide, such as impulsivity, effect the association between aggression and suicide (Gvion & Apter, 2011).

Future research should examine which facets of social support are most important in understanding the connection between aggression and suicide attempts. Arguably, our measure of social networks largely assessed emotional support from others – three of the four questions on this measure are related to the number of people one feels close to or can rely on. It remains unknown how other forms of social support (e.g. practical/instrumental support, informational support) impact the association between aggression and suicide.

The current study's results also imply that interventions aimed at mitigating suicide risk within this population may benefit from directly targeting aggressive behavior. Such a suggestion is especially relevant to suicide prevention within military populations given that aggression may be viewed as a highly valued, adaptive behavior in the military (Morland, Love, Mackintosh, Greene, & Rosen, 2012). Aggression may also serve as a marker for future suicide risk among active-duty service members. Further, results of our study suggest that early identification and intervention on problematic aggression may mitigate loss of social connection and frequent alcohol use, two factors already widely addressed in current suicide prevention efforts (Hou et al., 2022; Jin, Khazem, & Anestis, 2016).

### Limitations and future directions

Evaluation of the present results necessitates discussion of the study's limitations. First, our measures were limited to assessments of alcohol use frequency, emotional social support, access to firearms in the home, and suicide attempts. Future research would benefit from a more comprehensive assessment of such variables, including consequences from alcohol use, other facets of social support, access to firearms outside the home, and other forms of suicide behaviors. It is possible that the behaviors examined herein (e.g. suicide attempts, aggressive behavior) could be conceived as unsuitable for military service and thus likely underreported by service members due to denial, stigma, and/or over-punishment for mental health problems (Guina, Welton, Broderick, & Peirson, 2018; Palamar, Martins, Su, & Ompad, 2015). Relatedly, our measure of social support did not assess perceived loneliness or identify who participants felt close to. As noted earlier, these data do not identify those who died from a suicide attempt, which may have attenuated the association between firearm ownership and suicide attempts. Third, our sample was comprised of Army soldiers transitioning from pre- and post-deployment, limiting generalizability of our results to other military branches. Fourth, as with any longitudinal study, we observed missing data during follow-up assessments. Although we used an imputation method appropriate for our data, imputation can only use information from observed data. Thus, our results may not accurately reflect associations only observed in the missing data.

## Conclusion

Aggression has been identified as a potent risk factor for suicide in civilian populations, and our study adds to the small but growing body of research underscoring the significance of aggression in understanding military suicide. The present study also represents one of the first efforts to identify mechanisms of this association and highlights the roles of social networks and alcohol use frequency as mediators of the relation between aggression and suicide among Army soldiers. Understanding the role of aggression can inform targeted suicide prevention interventions aimed at reducing suicide risk among service members, an essential task in the face of rising suicide rates among military personnel.

## References

[ref2] Allen, J. J., & Anderson, C. A. (2017). Aggression and violence: Definitions and distinction. In P. Sturmey (Ed.), The Wiley handbook of violence and aggression (1st ed., pp. 1–14). Chichester, UK: Wiley. 10.1002/9781119057574.whbva001

[ref3] Bohnert, K. M., Ilgen, M. A., Louzon, S., McCarthy, J. F., & Katz, I. R. (2017). Substance use disorders and the risk of suicide mortality among men and women in the US Veterans Health Administration. Addiction, 112(7), 1193–1201. 10.1111/add.13774 28301070

[ref4] Calati, R., Ferrari, C., Brittner, M., Oasi, O., Olié, E., Carvalho, A. F., & Courtet, P. (2019). Suicidal thoughts and behaviors and social isolation: A narrative review of the literature. Journal of Affective Disorders, 245, 653–667. 10.1016/j.jad.2018.11.022 30445391

[ref5] Chu, C., Zuromski, K. L., Bernecker, S. L., Gutierrez, P. M., Joiner, T. E., Liu, H., … Nock, M. K. (2020). A test of the interpersonal theory of suicide in a large, representative, retrospective and prospective study: Results from the Army Study to Assess Risk and Resilience in Servicemembers (Army STARRS). Behaviour Research and Therapy, 132, 103688. 10.1016/j.brat.2020.103688 32731055 PMC10351027

[ref6] Clare, C. A., Velasquez, G., Mujica Martorell, G. M., Fernandez, D., Dinh, J., & Montague, A. (2021). Risk factors for male perpetration of intimate partner violence: A review. Aggression and Violent Behavior, 56, 101532. 10.1016/j.avb.2020.101532

[ref7] Coccaro, E. F., Fridberg, D. J., Fanning, J. R., Grant, J. E., King, A. C., & Lee, R. (2016). Substance use disorders: Relationship with intermittent explosive disorder and with aggression, anger, and impulsivity. Journal of Psychiatric Research, 81, 127–132. 10.1016/j.jpsychires.2016.06.011 27442963 PMC5744873

[ref9] Conner, K. R., Duberstein, P. R., Conwell, Y., & Caine, E. D. (2003). Reactive aggression and suicide: Theory and evidence. Aggression and Violent Behavior, 8(4), 413–432. 10.1016/S1359-1789(02)00067-8

[ref10] Conner, A., Azrael, D., & Miller, M. (2019). Suicide case-fatality rates in the United States, 2007 to 2014: A nationwide population-based study. Annals of Internal Medicine, 171(12), 885. 10.7326/M19-1324 31791066

[ref8] Conner, K. R., & Ilgen, M. A. (2011). Substance use disorders and suicidal behaviour. In R. C. O'Connor, S. Platt, & J. Gordon (Eds.), The international handbook of suicide prevention: Research, policy and practice (pp. 93–107). Chichester, UK: Wiley Blackwell.

[ref11] Department of Defense. (2022). Annual report on suicide in the military calendar year 2022. https://www.dspo.mil/Portals/113/Documents/ARSM_CY22.pdf

[ref48] Elbogen, E. B., Wagner, H. R., Kimbrel, N. A., Brancu, M., Naylor, J., Graziano, R., & Crawford, E., & VA Mid-Atlantic MIRECC Workgroup. (2018). Risk factors for concurrent suicidal ideation and violent impulses in military veterans. Psychological Assessment, 30(4), 425–435. 10.1037/pas0000490 28627921 PMC5736469

[ref12] Franklin, J. C., Ribeiro, J. D., Fox, K. R., Bentley, K. H., Kleiman, E. M., Huang, X., … Nock, M. K. (2017). Risk factors for suicidal thoughts and behaviors: A meta-analysis of 50 years of research. Psychological Bulletin, 143(2), 187–232. 10.1037/bul0000084 27841450

[ref13] Gromatsky, M., Edwards, E. R., Sullivan, S. R., van Lissa, C. J., Lane, R., Spears, A. P., … Goodman, M. (2022). Characteristics of suicide attempts associated with lethality and method: A latent class analysis of the Military Suicide Research Consortium. Journal of Psychiatric Research, 149, 54–61. 10.1016/j.jpsychires.2022.02.016 35231792

[ref14] Guina, J., Welton, R. S., Broderick, P. J., & Peirson, R. P. (2018). The military mental health disability system. In L. W. Roberts & C. H. Warner (Eds.), Military and veteran mental health (pp. 157–167). New York: Springer. 10.1007/978-1-4939-7438-2_11

[ref15] Gvion, Y. (2018). Aggression, impulsivity, and their predictive value on medical lethality of suicide attempts: A follow-up study on hospitalized patients. Journal of Affective Disorders, 227, 840–846. 10.1016/j.jad.2017.11.033 29689698

[ref16] Gvion, Y., & Apter, A. (2011). Aggression, impulsivity, and suicide behavior: A review of the literature. Archives of Suicide Research, 15(2), 93–112. 10.1080/13811118.2011.565265 21541857

[ref17] Hammett, J. F., Lavner, J. A., Karney, B. R., & Bradbury, T. N. (2021). Intimate partner aggression and marital satisfaction: A cross-lagged panel analysis. Journal of Interpersonal Violence, 36(3–4), NP1463–1481NP. 10.1177/0886260517747607 29295030 PMC10510459

[ref18] Hartley, C. M., Pettit, J. W., & Castellanos, D. (2018). Reactive aggression and suicide-related behaviors in children and adolescents: A review and preliminary meta-analysis. Suicide and Life-Threatening Behavior, 48(1), 38–51. 10.1111/sltb.12325 28044358 PMC7894982

[ref19] Hayes, A. F. (2022). Introduction to mediation, moderation, and conditional process analysis: A regression-based approach (3rd ed.). New York, NY, USA: The Guilford Press.

[ref20] Heinz, A. J., Cohen, N. L., Holleran, L., Alvarez, J. A., & Bonn-Miller, M. O. (2016). Firearm ownership among military veterans with PTSD: A profile of demographic and psychosocial correlates. Military Medicine, 181(10), 1207–1211. 10.7205/MILMED-D-15-00552 27753553 PMC5308415

[ref21] Hou, X., Wang, J., Guo, J., Zhang, X., Liu, J., Qi, L., & Zhou, L. (2022). Methods and efficacy of social support interventions in preventing suicide: A systematic review and meta-analysis. Evidence Based Mental Health, 25(1), 29–35. 10.1136/ebmental-2021-300318 34911688 PMC8788249

[ref22] Houtsma, C., Butterworth, S. E., & Anestis, M. D. (2018). Firearm suicide: Pathways to risk and methods of prevention. Current Opinion in Psychology, 22, 7–11. 10.1016/j.copsyc.2017.07.002 30122279

[ref23] Jin, H. M., Khazem, L. R., & Anestis, M. D. (2016). Recent advances in means safety as a suicide prevention strategy. Current Psychiatry Reports, 18(10), 96. 10.1007/s11920-016-0731-0 27629355

[ref24] Kearns, J. C., Edwards, E. R., Finley, E. P., Geraci, J. C., Gildea, S. M., Goodman, M., … Kessler, R. C. (2023). A practical risk calculator for suicidal behavior among transitioning U.S. Army soldiers: Results from the Study to Assess Risk and Resilience in Servicemembers-Longitudinal Study (STARRS-LS). Psychological Medicine, 53(15), 7096–7105. 10.1017/S0033291723000491 37815485 PMC10575670

[ref25] Kessler, R. C., Colpe, L. J., Fullerton, C. S., Gebler, N., Naifeh, J. A., Nock, M. K., … Heeringa, S. G. (2013a). Design of the Army Study to Assess Risk and Resilience in Servicemembers (Army STARRS): Army STARRS design. International Journal of Methods in Psychiatric Research, 22(4), 267–275. 10.1002/mpr.1401 24318217 PMC3992857

[ref26] Kessler, R. C., Santiago, P. N., Colpe, L. J., Dempsey, C. L., First, M. B., Heeringa, S. G., … Ursano, R. J. (2013b). Clinical reappraisal of the Composite International Diagnostic Interview Screening Scales (CIDI-SC) in the Army Study to Assess Risk and Resilience in Servicemembers (Army STARRS): Clinical reappraisal of the CIDI-SC in Army STARRS. International Journal of Methods in Psychiatric Research, 22(4), 303–321. 10.1002/mpr.1398 24318219 PMC4027964

[ref27] Klonsky, E. D., & May, A. M. (2015). The Three-Step Theory (3ST): A new theory of suicide rooted in the ‘ideation-to-action’ framework. International Journal of Cognitive Therapy, 8(2), 114–129. 10.1521/ijct.2015.8.2.114

[ref28] Krauss, A., Edwards, E. R., Ruiz, D., Epshteyn, G., Coolidge, B., & Goodman, M. (2023). Understanding changes in aggression among U.S. army soldiers: The role of trauma exposure during deployment. Journal of Psychiatric Research, 164, 202–208. 10.1016/j.jpsychires.2023.06.015 37356353

[ref29] Li, C.-H. (2016). Confirmatory factor analysis with ordinal data: Comparing robust maximum likelihood and diagonally weighted least squares. Behavior Research Methods, 48(3), 936–949. 10.3758/s13428-015-0619-7 26174714

[ref30] MacManus, D., Rona, R., Dickson, H., Somaini, G., Fear, N., & Wessely, S. (2015). Aggressive and violent behavior among military personnel deployed to Iraq and Afghanistan: Prevalence and link with deployment and combat exposure. Epidemiologic Reviews, 37(1), 196–212. 10.1093/epirev/mxu006 25613552

[ref31] McGlade, E., Bueler, E., DiMuzio, J., Sheth, C., Legarreta, M., & Yurgelun-Todd, D. (2021). Sex differences in suicidal behaviors and aggression in US Veterans. Psychiatry Research, 301, 113982. 10.1016/j.psychres.2021.113982 33993038

[ref32] Milner, A., Witt, K., Maheen, H., & LaMontagne, A. (2017). Access to means of suicide, occupation and the risk of suicide: A national study over 12 years of coronial data. BMC Psychiatry, 17(1), 125. 10.1186/s12888-017-1288-0 28376757 PMC5379531

[ref33] Morland, L. A., Love, A. R., Mackintosh, M., Greene, C. J., & Rosen, C. S. (2012). Treating anger and aggression in military populations: Research updates and clinical implications. Clinical Psychology: Science and Practice, 19(3), 305–322. 10.1111/cpsp.12007

[ref34] Office of the Surgeon General, United States Army Medical Command, Office of the Command Surgeon, HQ USCENTCOM, & Office of the Command Surgeon, U.S. Forces Afghanistan. (2011). Joint Mental Health Advisory Team 7 (J-MHAT7): Operation Enduring Freedom 2010 Afghanistan (p. 112). United States Army Medical Command.

[ref35] Oquendo, M. A., Galfalvy, H. C., Choo, T.-H., Kandlur, R., Burke, A. K., Sublette, M. E., … Stanley, B. H. (2021). Highly variable suicidal ideation: A phenotypic marker for stress induced suicide risk. Molecular Psychiatry, 26(9), 5079–5086. 10.1038/s41380-020-0819-0 32576966 PMC7755748

[ref36] Palamar, J. J., Martins, S. S., Su, M. K., & Ompad, D. C. (2015). Self-reported use of novel psychoactive substances in a US nationally representative survey: Prevalence, correlates, and a call for new survey methods to prevent underreporting. Drug and Alcohol Dependence, 156, 112–119. 10.1016/j.drugalcdep.2015.08.028 26377051 PMC4633323

[ref37] Poorolajal, J., Haghtalab, T., Farhadi, M., & Darvishi, N. (2016). Substance use disorder and risk of suicidal ideation, suicide attempt and suicide death: A meta-analysis. Journal of Public Health, 38(3), e282–e291. 10.1093/pubmed/fdv148 26503486

[ref38] Posner, K., Brown, G. K., Stanley, B., Brent, D. A., Yershova, K. V., Oquendo, M. A., … Mann, J. J. (2011). The Columbia–Suicide Severity Rating Scale: Initial validity and internal consistency findings from three multisite studies with adolescents and adults. American Journal of Psychiatry, 168(12), 1266–1277. 10.1176/appi.ajp.2011.10111704 22193671 PMC3893686

[ref39] Rosseel, Y. (2012). lavaan: An R package for structural equation modeling. Journal of Statistical Software, 48(2), 1–36. 10.18637/jss.v048.i02

[ref40] Rynar, L., & Coccaro, E. F. (2018). Psychosocial impairment in DSM-5 intermittent explosive disorder. Psychiatry Research, 264, 91–95. 10.1016/j.psychres.2018.03.077 29627702 PMC5983894

[ref41] Sanchez, C., Jaguan, D., Shaikh, S., McKenney, M., & Elkbuli, A. (2020). A systematic review of the causes and prevention strategies in reducing gun violence in the United States. The American Journal of Emergency Medicine, 38(10), 2169–2178. 10.1016/j.ajem.2020.06.062 33071102

[ref42] Schafer, K. M., Duffy, M., Kennedy, G., Stentz, L., Leon, J., Herrerias, G., … Joiner, T. E. (2022). Suicidal ideation, suicide attempts, and suicide death among Veterans and service members: A comprehensive meta-analysis of risk factors. Military Psychology, 34(2), 129–146. 10.1080/08995605.2021.1976544 38536290 PMC10013359

[ref43] Sherman, H., Frye-Cox, N., & Lucier-Greer, M. (2023). Combat deployment experiences and soldier mental health: Examining the factor structure of a Combat Experiences Scale. Military Medicine, 188(5–6), e1156–e1165. 10.1093/milmed/usab456 34755866

[ref44] Silverberg, N. D., Iverson, G. L., Cogan, A., Dams-O-Connor, K., Delmonico, R., Graf, M. J. P., … … Zemek, R. (2023). The American Congress of Rehabilitation Medicine diagnostic criteria for mild traumatic brain injury. Archives of Physical Medicine and Rehabilitation, 104(8), 1343–1355. 10.1016/j.apmr.2023.03.036 37211140

[ref45] Sokol, Y., Gromatsky, M., Edwards, E. R., Greene, A. L., Geraci, J. C., Harris, R. E., & Goodman, M. (2021). The deadly gap: Understanding suicide among veterans transitioning out of the military. Psychiatry Research, 300, 113875. 10.1016/j.psychres.2021.113875 33901974

[ref46] Start, A. R., Allard, Y., Adler, A., & Toblin, R. (2019). Predicting suicide ideation in the military: The independent role of aggression. Suicide and Life-Threatening Behavior, 49(2), 444–454. 10.1111/sltb.12445 29498089

[ref47] Stekhoven, D. J., & Bühlmann, P. (2012). Missforest – Non-parametric missing value imputation for mixed-type data. Bioinformatics, 28(1), 112–118. 10.1093/bioinformatics/btr597 22039212

[ref1] U.S. Department of Veterans Affairs, Office of Mental Health and Suicide Prevention. (2023). 2023 national veteran suicide prevention annual report. https://www.mentalhealth.va.gov/docs/data-sheets/2023/2023-National-Veteran-Suicide-Prevention-Annual-Report-FINAL-508.pdf

[ref49] Van Orden, K. A., Witte, T. K., Cukrowicz, K. C., Braithwaite, S. R., Selby, E. A., & Joiner, T. E. (2010). The interpersonal theory of suicide. Psychological Review, 117(2), 575–600. 10.1037/a0018697 20438238 PMC3130348

[ref50] Vogt, D., Smith, B. N., King, L. A., King, D. W., Knight, J., & Vasterling, J. J. (2013). Deployment Risk and Resilience Inventory-2 (DRRI-2): An updated tool for assessing psychosocial risk and resilience factors among service members and veterans. Journal of Traumatic Stress, 26(6), 710–717. 10.1002/jts.21868 24490250

[ref51] Watkins, L. E., Sippel, L. M., Pietrzak, R. H., Hoff, R., & Harpaz-Rotem, I. (2017). Co-occurring aggression and suicide attempt among veterans entering residential treatment for PTSD: The role of PTSD symptom clusters and alcohol misuse. Journal of Psychiatric Research, 87, 8–14. 10.1016/j.jpsychires.2016.12.009 27984702

[ref52] Wilkins, K. C., Lang, A. J., & Norman, S. B. (2011). Synthesis of the psychometric properties of the PTSD checklist (PCL) military, civilian, and specific versions. Depression and Anxiety, 28(7), 596–606. 10.1002/da.20837 21681864 PMC3128669

